# Band 3 Missense Mutations and Stomatocytosis: Insight into the Molecular Mechanism Responsible for Monovalent Cation Leak

**DOI:** 10.1155/2011/136802

**Published:** 2011-08-23

**Authors:** Damien Barneaud-Rocca, Bernard Pellissier, Franck Borgese, Hélène Guizouarn

**Affiliations:** ^1^Institut de Biologie du Développement et Cancer, UMR6543, CNRS, 28 Avenue Valrose, 06108 Nice Cedex 2, France; ^2^Institut de Biologie du Développement et Cancer, Université de Nice, 06108 Nice Cedex 2, France

## Abstract

Missense mutations in the erythroid band 3 protein (Anion Exchanger 1) have been associated with hereditary stomatocytosis. Features of cation leaky red cells combined with functional expression of the mutated protein led to the conclusion that the AE1 point mutations were responsible for Na^+^ and K^+^ leak through a conductive mechanism. A molecular mechanism explaining mutated AE1-linked stomatocytosis involves changes in AE1 transport properties that become leaky to Na^+^ and K^+^. However, another explanation suggests that point-mutated AE1 could regulate a cation leak through other transporters. This short paper intends to discuss these two alternatives.

## 1. Introduction

Band 3 or anion exchanger 1 (AE1) is the major red cell membrane protein. It belongs to the Solute Carrier 4A family (SLC4A) grouping bicarbonate transporters [1–3]. This protein catalyzes electroneutral chloride-bicarbonate exchange, and it is also expressed in kidney *α*-intercalated cells and in cardiomyocytes [4, 5]. In red cells, it is involved in two main tasks: enhancement of carbon dioxide transport and structuration of cell shape. It is found in red cells from all vertebrates except lampreys which naturally do not express erythrocyte AE1 [6]. Besides this exception, its complete absence from mammalian red cells leads to red cell defects whose consequences on health depend on the species. Dyserythropoiesis, severe haemolytic anaemia, and often premature death have been reported in mouse [7], and human [8], whereas cow or zebra fish seems to better withstand red cell AE1 deficiency [9, 10]. 

In human, many different mutations in *SLC4A1* gene coding for AE1 have been reported [11]. Some of them are asymptomatic, whereas some others are associated with red cell pathologies characterized by alteration of red cell shape and rheological properties. As this protein is also expressed in kidney, a renal phenotype can be associated with *SLC4A1* mutations. In this paper we will focus on red cell AE1, and the reader interested in kidney AE1 is therefore addressed to very exhaustive recent reviews on this subject [12–15]. 

When a red cell phenotype is associated with *SLC4A1* mutations, the symptoms are hyperhaemolysis and anaemia, icterus, and splenomegaly. However, these symptoms may vary widely in intensity. It appears that the *SLC4A1* mutations can be divided into two classes according to the way they impair AE1 function: (1) those that prevent correct folding of the protein so that it is not addressed to plasma membrane. This leads to a lower amount of AE1 in red cell membrane that impairs connection of skeleton and membrane, a feature of hereditary spherocytosis condition [16, 17]; (2) those that are associated with an increased cation permeability of red cell membrane. This latter condition is the hallmark of hereditary stomatocytosis [18, 19].

Since the initial discovery that 5 point mutations in *SLC4A1* gene (responsible for L687P, D705Y, S731P, H734R, or R760Q substitutions in AE1) were associated with increased red cell Na^+^ and K^+^ leak [20], 4 other point mutations associated with similar red cell phenotype have been reported (G796R, E758K, S762R, and R730C) [21–24]. It has been proposed that the molecular mechanism accounting for cation leaky red cells in these hereditary stomatocytoses was a change in AE1 transport properties induced by the point mutations. The exchanger itself mediates cation leak by a conductive mechanism [25]. However, this interpretation leads to dramatically changing our way of thinking about band 3 transport mechanism. Moreover, the transport features of some of these AE1 mutants lead to another interpretation, that is, cation leak is due to the activation of endogenous Na^+^ and K^+^ transporters (or channels) in red cell membrane by mutated AE1 [23, 24, 26]. This short paper intends to discuss the molecular mechanism of red cell cation leak associated with AE1 point mutations in hereditary stomatocytosis.

### 1.1. Position of Amino Acid Substitutions in AE1 Polypeptide

AE1 polypeptide can be divided into 3 functional domains: a cytoplasmic amino-terminal domain, about 400 amino acids, interacting in red cells with various enzymes, haemoglobin, ankyrin, and band 4.2; a membrane spanning domain where anion exchange takes place and a short carboxy terminal end in the cytoplasm that associates with carbonic anhydrase II [27]. The protein forms part of a macrocomplex, combining membrane, and cytoplasmic proteins that are thought to improve the efficiency of gas transport by red cells [28]. 

**Figure 1:**
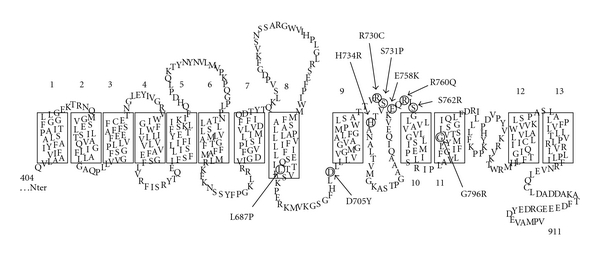
Topology of the membrane spanning domain of human AE1 with 13 *α* helices, according to Zhu et al. [29]. Aminoacids that are substituted in response to the different point mutations in AE1 gene are labelled by circles. Each of these mutations is associated with hereditary haemolytic anaemia characterized by cation leaky red cells.

Figure 1 illustrates the position of each of the point mutations that have been identified in patients with cation leaky red cells. The substitutions concern highly conserved amino acids among known electroneutral anion exchangers (SLC4A1, A2, and A3), and they are all located in the membrane spanning domain.

### 1.2. Point-Mutated AE1 and Permeability Features

The permeability of red cells from patients bearing heterozygous mutation on AE1 has been investigated, and the transport features of the point-mutated AE1 have been studied by expression in amphibian oocytes. Thus, in most cases, it was possible to combine data from red cells to data from heterologous expression system. These data are presented here and will be discussed in the third part.  Table 1 summarized the main features of cation leaky red cells and mutated AE1.

The red cell leaks Na^+^ and K^+^ by an ouabain- and bumetanide-resistant mechanism that is temperature dependent; it is increased by temperatures below 37°C. This has been extensively studied in red cells of patients heterozygous for L687P, D705Y, S731P, H734R, R760Q, and S762R AE1 mutants [20, 21]. The diffusion of K^+^ and Na^+^ according to their electrochemical gradients leads to osmotic fragility of the red cells. At body temperature, the cation leak can be more or less compensated depending on the mutants. An artefactual rise in plasma K^+^ (pseudohyperkalaemia) can be observed after cooling blood to room temperature [30]. The shape of cation leak temperature dependence is not identical between the mutants [20], and the morphology of red cells also shows some differences between patients: blood smears exhibit stomatocytes or spherocytes. In red cells with H734R or G796R AE1 mutations, an increased activity of Na^+^-K^+^-2Cl^−^ cotransporter, K^+^-Cl^−^ cotransporter, Na^+^/H^+^ exchanger, or K^+^/Na^+^/H^+^ exchanger has been reported [22, 26]. R730C mutant is also associated with increased activity of Na^+^/H^+^ exchanger and Na^+^/K^+^-pump, whereas the Gardos channel fluxes are reduced [23]. Thus, the red cell permeability results from both the monovalent cation leak induced by AE1 point mutations and the activity of solute carriers that have been stimulated by the initial Na^+^ and K^+^ movements. The regulation of these other carriers could differ between patients as well as how the body cope with cation leaky red cells. This could explain variations in patient's phenotypes (Table 1). 

For all the studied patients, the abundance of AE1 in red cells is grossly normal. However, the anion permeability of these cells is decreased suggesting a loss of anion exchange function of the protein [20]. Indeed, functional characterization of L687P, D705Y, R730C, S731P, H734R, S762R, and G796R mutants expressed in xenopus oocytes shows that they are no more able to exchange Cl^−^ and HCO_3_^−^ [21, 25]. In contrast E758K and R760Q mutants keep an anion exchange activity [24, 31]. Another interesting difference for these two mutants is that their abundance in plasma membrane is highly dependent on glycophorin A (GPA) coexpression. GPA is known to bind AE1 and to act as a chaperone. Moreover this interaction stimulates AE1 transport activity [32–34]. 

Very few studies are available about conductance of these stomatocytic red cells. Only conductance of red cells from two patients with R730C or H734R mutation on AE1 has been reported. Patch current recordings on 3 red cells from a patient with R730C AE1 mutation do not allow to detect increased cation conductance compared to normal red cells [23]. Similar conclusions have been drawn from conductance analyses on red cells with H734R AE1 mutation [26].

Expression of L687P, D705Y, S731P, H734R, R760Q, S762R, or G796R AE1 mutants in xenopus oocytes induces a reversal in xenopus oocyte Na^+^ and K^+^ contents after 3 days in medium with ouabain- and bumetanide. This cation leak is associated with increased ouabain and bumetanide-resistant Rb^+^ or Li^+^ uptake which is similar to the red cell cation leak [20–22]. The Na^+^ and K^+^ transport associated with AE1 missense mutations shows independent movement of Na^+^ and K^+^ with a 1 for 1 stoichiometry. Moreover, when assessed, a conductance has been associated with these cation movements. Thus the molecular mechanism responsible for the observed cation movement is a channel-like transport mechanism [25]. 

**Figure 2:**
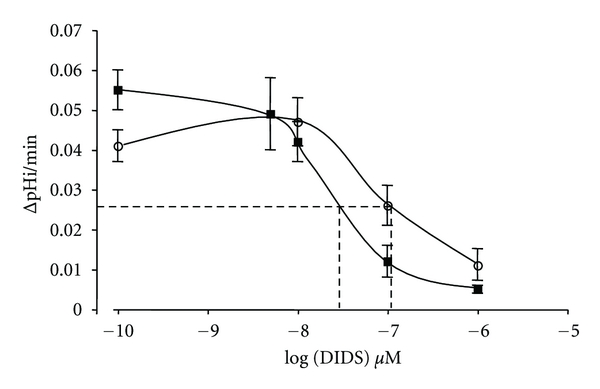
Dose response curves of Cl^−^/HCO_3_^−^ exchange by DIDS. The capacity of oocytes expressing wt AE1 or R760Q mutant (10 ng cRNA coinjected with 2.5 ng GPA-cRNA in both cases) to alkalinize in CO_2_/HCO_3_^−^ buffer without extracellular Cl^−^ (gluconate medium) was assessed in presence of different DIDS concentrations. The initial slope of alkalinization as a function of time was plotted against DIDS concentrations. The method used was described in a previous publication [25]. Data are means +/− s.e.m. of 9 to 20 oocytes from different batches. Black symbols correspond to wt AE1 expressing oocytes, empty circles to R760Q mutant expressing oocytes.

Pharmacology of transport activities of the mutants have been assessed in red cells or in heterologous expression systems. DIDS (4,4′-diisothiocyanatostilbene-2,2′,-disulfonate) has long been known to inhibit anion exchange at micromolar concentrations [35, 36]. Moreover, sulfonate radical of DIDS can link two different lysines (K539 and K851) in putative transmembrane helices (TM) 5 and 12 of AE1 membrane spanning domain [36]. It is thus possible to covalently bind DIDS on each or both of these two lysines. The DIDS derivative SITS (4-acetamido-4′-isothiocyano-2,2′-stilbene disulfonate), flufenamic acid, and niflumic acid are also potent inhibitors of AE1 activity. It is observed that the point mutations impair the protein sensitivity to classical anion exchanger inhibitors. For instance, S731P mutation prevents DIDS covalent binding to the exchanger [20]. For the two mutants keeping anion exchange activity, the DIDS sensitivity of the transport is also impaired.  Figure 2 illustrates Cl^−^/HCO_3_^−^ exchange as a function of DIDS concentrations for R760Q mutant compared to wt AE1. The R760Q mutation decreases AE1 DIDS sensitivity as shown by the right shift of the dose-response curve. The anion uptake mediated by E758K mutant is also less sensitive to DIDS than wtAE1 [24].

Pharmacology of the cation leak induced by AE1 point mutations has also been investigated. Inhibition of the Na^+^ and K^+^ leak induced by L687P, D705Y, S731P, H734R, and R760Q mutations has been observed in red cells with SITS, dipyridamole, and NS1652 also known to block anion exchanger [20]. Pharmacology of the cation leak is difficult to assess in xenopus oocytes since endogenous cation permeabilities could be activated by common AE1 inhibitors such as DIDS or niflumic acid [37, 38]. However, inhibition of the Na^+^ and K^+^ conductance induced by expression of mutated AE1 was observed with SITS and plurivalent cations such as Zn^2+^, La^3+^, and Gd^3+^ [24, 25].

Amongst AE1 point mutations associated with cation leaky red cells, two mutants (R730C and E758K) exhibit peculiar transport features in amphibian oocytes. R730C AE1 mutant induces only a weak ouabain- and bumetanide-resistant cation leak in xenopus oocytes. It is not possible to measure a significant increase in ouabain- and bumetanide-resistant ^86^Rb^+^ uptake, and only a *≈*2.5-fold increase in Li^+^ uptake is observed (to compare to *≈*8-fold increase in oocytes expressing S731P mutant for instance). Moreover expression of R730C mutant is associated with an increased activity of the Na^+^/K^+^ ATPase [23]. For E758K, mutant permeability features depend on the expression system. It has been studied in two different amphibian oocytes, xenopus, and ambystoma. In both species, its abundance in plasma membrane is dependent on the coexpression of GPA. The mutant keeps anion exchange activity and induces a ^86^Rb^+^ uptake in both systems. However, it appears that this Rb permeability is not correlated to the expression level of the mutant when expressed in ambystoma: the higher number of transporters when coexpressed with GPA does not induce a higher ^86^Rb uptake. The expression of E758K mutant also slightly increases xenopus oocyte conductance, but this conductance does not account for the observed Rb^+^ permeability as deduced from differences in pharmacological pattern [24].

### 1.3. What Is the Pathway for Cations in Cells Expressing Point Mutated AE1?

Point mutations in AE1 are associated with monovalent cation leak in red cells as in heterologous expression systems. This monovalent cation leak could be correlated to a nonselective cation conductance and to elevated activity of endogenous transport systems.

Two possibilities that are not exclusive could be envisioned: the missense mutations in AE1 polypeptide change the transport properties of the protein that becomes leaky to Na^+^ and K^+^, or the mutated AE1 stimulates native transporters for Na^+^ and K^+^ in red cells as in heterologous expression systems.

The work on trout AE1 has shown that this protein could interact with Na^+^-K^+^-2Cl^−^ cotransporter by its carboxy terminal domain, stimulating the activity of this transporter in xenopus oocytes [39]. Reports on E758K and R730C AE1 mutations suggest that the cation leak associated with these mutations likely involves activation of still undefined endogenous transporters. 

As AE1 forms part of a macrocomplex, it functionally interacts with carbonic anhydrase [27, 40], and it is also involved in many molecular interactions in red cells, with ankyrin, glycophorin A (GPA), glycolytic enzymes, or haemoglobin, for instance [28, 41–43]. It is thus plausible that point-mutated AE1 could interfere with different endogenous transporters (understood as pumps, channels, or carriers) in red cells as in heterologous expression systems. It could be proposed that point mutations by changing AE1 conformation enable molecular interactions regulating the activity of various endogenous transporters. An AE1 mutated conformation could be envisioned which would not dramatically change AE1 transport features but would activate endogenous monovalent cation permeabilities in red cells as in heterologous expression systems. That should happen with different point mutations in AE1 membrane spanning domain. 

Since pioneer work of electrophysiologists on red cells in the 80s [44], numerous cation and anion conductances have been characterized. Anion conductances (maxianion channels [45, 46]) or cation conductances such as nonselective Ca^2+^ permeable cation channels (L-type Ca^2+^ channel, voltage-dependent) or nonselective voltage-independent cation channels (NSVCCs) [47–51] and Ca^2+^ sensitive K^+^ channel (Gardos channel) [44] are well characterized in human red cells. It has been proposed that the TRPC6, member of the Transient Receptor Potential family proteins, contributes to the nonselective voltage-independent cation current in red cells [52]. However, the molecular identity of channels responsible for most of the electrophysiologically described conductances is unknown. It could be proposed that the nonselective Na^+^ and K^+^ leak induced by AE1 point mutations could be mediated by one of these conductances. However, the features of the cation leak associated with AE1 point mutations do not point out any of the red cell channels described so far. In particular, this cation leak is insensitive to amiloride, known to block the NSVCC; it is insensitive to extracellular Cl^−^ concentration, known to stimulate red cell cation channels; it is insensitive to extracellular Ca^2+^ concentration [25]. Thus, in red cells as in heterologous expression system, the molecular identity of the transporters eventually activated by point-mutated AE1 still remains unknown as their activating mechanism. 

Whatever the origin of the cation leak induced by AE1 point mutations, the consecutive alteration of cation permeability will impair red cell homeostasis and modulate the activity of different transporters. The permeability features of red cells with H734R, G796R, or R730C AE1 mutations show that the activity of different transporters could be stimulated: Na^+^-K^+^-2Cl^−^ cotransporter, Na^+^/K^+^ ATPase, Na^+^(K^+^)/H^+^ exchanger, and K^+^-Cl^+^ cotransporter [22, 23, 26]. This could be due to the fact that point mutated AE1 induced a cation leak locally changing cation concentrations. These changes could be responsible for subsequent activation of various cation transporters. As a consequence, a same AE1 mutation could lead to diverse phenotypes in red cells depending on how the endogenous transporters react to the initial cation leak. 

In the absence of identified native transporter mediating the cation leak associated with AE1 point mutations, the hypothesis of a cation leaky AE1 is challenging and the simplest to propose. Moreover, it provides an attractive approach to understand the transport mechanism of this protein. Whereas it is depicted as a typical electroneutral anion exchanger, the anion exchange rate through AE1 is extremely fast (10 000 per second) and Cl^−^ slippage occurs occasionally (1 for 10 000 exchange). Crystallographic structures are not available yet with enough resolution to help understand the transport mechanism [53]. This mechanism should allow very rapid conformational changes that could resemble alternately opened gates for instance. Previous work on trout AE1 has shown that this exchanger can behave as an anion conductance permeable to organic solutes (taurine, sorbitol) and to monovalent cations (Na^+^ and K^+^) [54, 55]. The work on a truncated human AE1 has also shown that this protein could behave like a conductance when it was deleted of transmembrane segments 6 and 7 [56]. Thus, it appears feasible to convert the electroneutral anion exchanger into a conductive pathway by different manœuvres. Former studies in red cells, based on pharmacological evidence, have also suggested that a monovalent cation leak through AE1 could be induced by decreasing extracellular Cl^−^ concentration [57]. The speculations about a monovalent cation leak through AE1 polypeptide have to combine the following considerations.

**Figure 3:**
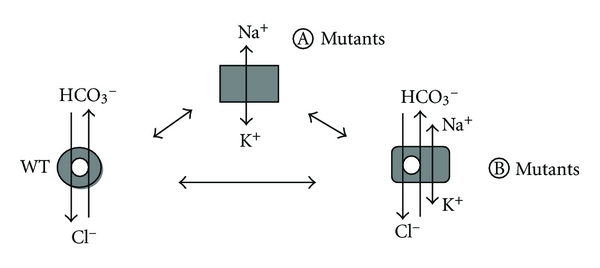
Putative transport states of AE1. Whereas the wt AE1 does only exchange Cl^−^ and HCO_3_^−^, a *A* mutated state only conducts monovalent cations and a *B* mutated state shows both transport activities, anion exchange and cation leak. The *A* state should be obtained with L687P, D705Y, R730C, S731P, H734R, S762R and G796R mutations. The *B* state should be obtained with E758K and R760Q mutations.

The point mutations induce a conformational change of the protein as suggested by changes in pharmacology sensitivity (Figure 2), by impairment of anion exchange capacity, or by requirement of GPA for correct addressing to plasma membrane. Moreover, diverse point mutations are likely to produce a similar AE1 conformation as deduced from similar permeability patterns. The transport features of cells expressing AE1 suggest at least three functional states for AE1: the wt state, a *A* mutated state where no anion exchange occurs only cation leak exists and a *B* mutated state where anion exchange and cation leak coexist (Figure 3). Whether or not these functional states are linked to different structural states of AE1 has to be shown but it is likely.

It appears that apparent unrelated positions in AE1 membrane spanning domain are susceptible to impair anion exchange function in the same way: point mutations L687P, D705Y, S731P, H734R, S762R, and G796R abolish anion exchange and induce a similar monovalent cation leak that is also similar to the cation leak observed in cells expressing the mutated AE1 responsible for South East Asian Ovalocytosis (SAO AE1) [21]. SAO AE1 is deleted of 9 amino acids at the junction of cytoplasmic domain and membrane spanning domain of the protein [58]. This mutation is prevalent in South East Asian population where it is proposed to protect against severe forms of malaria [59]. Thus, the cation leaky conformation appears as a complex organization involving distant segments in the membrane spanning domain of the protein, and this conformation could be obtained either by a deletion at the junction of cytoplasmic and transmembrane domain (SAO AE1) or by some specific point mutations. The cytoplasmic domain, *per se*, is probably not involved in the cation leaky conformation as suggested by the work on a trout AE1 deleted of cytoplasmic domain that kept its conductive transport mechanism [60].

In wtAE1, the anion exchange site involves transmembrane helix 8 (TM 8) and an anion selectivity filter including a region at the top of TM 12 and 13 and amino acids in the loop connecting TM 7 and 8 [61, 62]. Our recent work on the cation leaky H734R mutant has shown that the same TM 8 was also involved in cation movement suggesting a common pathway for anions and cations through AE1 [63]. Moreover, it was shown that amino acids in the intracellular loop connecting TM 8 and TM 9 play an important role in AE1 transport features. For instance, substitution of the charged residues in this loop induces a cation leak and severely impairs anion exchange activity. The position of the point mutations S731P, H734R, E758K, R760Q, and S762R at both extremities of the next loop connecting TM 9 and 10 also suggests an important functional role for this central part of the membrane spanning domain. Amino acid substitutions in these two loops could change the orientation, rotation, or movements of TM 8, 9, and 10 and impair AE1 transport site. The leak could be seen as a broken seal in the transport system that leaks Na^+^ and K^+^ for which a high driving force exists. This leads to consider that the transport site is susceptible to structural changes that could be induced by diverse but specific amino acid substitutions. This change unmasks a conductance for monovalent cations that does not seem to interfere with the ability to exchange anions since some mutants exhibit both transport activities.

The possibility for a carrier to function as a channel seems conflicting. Indeed channels are seen as structures that could simultaneously connect intra- and extracellular medium, what should never happen through a carrier. However, there are increasing examples of membrane proteins with ambiguous behaviour between channels and transporters. A historical example of transporter with channel activity is the glutamate transporter which is also a chloride channel [64–66]. Chloride channels, Na^+^-K^+^ pump, are other examples of ambiguous transport mechanisms between channels and transporters which strengthen our simple hypothesis of cation leaky AE1 [65–69]. In red cells, monovalent cation leak has also been associated with heterozygous mutations on RhAG (Rhesus Associated Glycoprotein) gene. Two different amino acid substitutions in RhAG could turn on a cation pore through this membrane protein proposed to be a NH_3_/NH_4_^+^ transporter in red cells [70].

## 2. Conclusion

Whereas specific AE1 mutations are undoubtedly linked to cation leaky red cells responsible for hereditary haemolytic anaemia, it is observed that all the 9 AE1 mutations presented here do not impair AE1 transport features in a similar manner. Moreover, membrane permeability of cells expressing point-mutated AE1 shows some differences suggesting a complicated regulation of this permeability. The proposition of point mutations altering AE1 transport mechanism is an attractive hypothesis supported by experimental evidence. However, this does not exclude the possibility for some mutated AE1 to also regulate the activity of other transporters. 

Resolution of the 3D structure of AE1 would greatly help to understand the peculiar transport properties of this surprising protein. It would be of particular interest to know how the studied AE1 point mutations alter AE1 structure, if these different point mutations have a common mechanism of action on the structure. A better understanding of the mechanism of interactions between AE1 and its partners would also help to assign new regulatory function to AE1.

## Figures and Tables

**Table 1:** Features of cation leaky red cells and point-mutated AE1.

AE1 point mutation	Red cell shape	Abundance of AE1 (band 3) in red cell membrane^1^	Red cell cation leak rate at 0°C (multiple of normal)^1^	Anion exchange activity of mutated AE1^2^	Pharmacology of the red cell cation leak	Pharmacology of the cation leak in heterologous expression system (cation conductance or cation flux)
L687P	Stomatocyte	82%	7-8	Abolished	NS1652, SITS, dipyridamol	Cation conductance: SITS, Zn^2+^, La^3+^ sensitive
D705Y	Spherocyte	77%	8	Abolished	NS1652, SITS, dipyridamol	Cation conductance: SITS, Zn^2+^, La^3+^ sensitive
R730C	Stomatocyte	Normal	6 (at 37°C)	Abolished	NT	NT
S731P	Stomatocyte	79%	30–57-58–87	Abolished	NS1652, SITS, dipyridamol	Cation conductance: SITS, Zn^2+^, La^3+^ sensitive
H734R	Stomatocyte	74–82%	87–94	Abolished	NS1652, SITS, dipyridamol	Cation conductance: SITS, Zn^2+^, La^3+^ sensitive
E758K	Spherostomatocyte	Mild deficiency	NT	Normal with GPA coexpression	NT	Rb flux: DIDS, Zn^2+^, Gd^3+^ sensitive. Conductance: Zn^2+^, SITS and WW-781 sensitive
R760Q	Spherocyte	85–92%	4–6	74% of wt (with GPA)	NS1652, SITS, dipyridamol	NT
S762R	Stomatocyte	NT	7	Abolished	NT	NT
G796R	Stomatocyte	Normal	NT	Abolished	NT	Li uptake insensitive to SITS or H_2_DIDS

This table summarized data collected from different publications. For L687P, D705Y, S731P, H734R, [20, 25]. For R730C, [23]. For E758K, [24]. For R760Q, [20, 31]. For S762R, [21]. For G796R, [22]. NT: not tested.

^1^Each number refers to features of red cells from different patients carrying the same AE1 mutation.

^2^The anion exchange was assessed in amphibian oocyte expressing the mutated AE1. The loss of anion exchange is confirmed in heterozygote red cells by *≈*50% decreased anion flux.
